# Cuttings from adult African mahogany individuals through tissue rejuvenation/reinvigoration

**DOI:** 10.1007/s13205-025-04463-7

**Published:** 2025-08-08

**Authors:** Cassia dos Santos Azevedo, Júlio Cézar Tannure Faria, Marcos Vinicius Winckler Caldeira, Tiago de Oliveira Godinho, Heloisa Oliveira dos Santos, Wilson Vicente Souza Pereira, Gabriel Soares Lopes Gomes, Dione Richer Momolli, Cristiane Coelho de Moura, Laura Ribeiro Atala

**Affiliations:** 1 Department of Forest and Wood Sciences, Federal University of Espírito Santo, Alegre, Espirito Santo Brazil; 2 Department of Environmental Sciences, Federal University of São Carlos, Sorocaba, São Paulo 18052-780 Brazil; 3 Vale Nature Reserve (RNV), BR 101N, Linhares, Espírito Santo 29911-080 Brazil; 4 Department of Agronomy, Federal University of Lavras, Lavras, Minas Gerais 37200-900 Brazil; 5 Department of Forest Sciences, Federal University of Lavras, Lavras, Minas Gerais 37200-900 Brazil

**Keywords:** *Khaya* spp., Vegetative propagation, Adventitious rooting, Antioxidant enzymes, Proteins, Carbohydrates

## Abstract

Most woody species undergo morphological, physiological, and biochemical changes during ontogenetic development from juvenile to adult phases, especially in terms of clonal potential, growth vigor, and adventitious rooting capacity. This study focused on generating information to support the vegetative propagation of adult individuals of *Khaya* spp., aiming to optimize the mechanisms involved in the induction of adventitious rooting and its association with indole-3-butyric acid (IBA). The experiment evaluated the rejuvenation/reinvigoration of plant tissues from three *Khaya* species by comparing juvenile and adult materials, epicormic shoots, and tissues rescued from the base of felled trees. In contrast to most studies, which focus primarily on juvenile materials, this is the first to investigate cloning through the rescue of adult genotypes, targeting the multiplication of individuals expressing superior phenotypic traits in the field. The results indicated that IBA application did not significantly influence survival, rooting, or the occurrence of abnormalities in the cuttings. Although adventitious rooting was achieved for all species, the percentages did not exceed 30%, reflecting typical results for species with low propagation potential. Basal cuttings showed enzymatic activities of superoxide dismutase, catalase, and peroxidase similar to those observed in juvenile materials, although total protein and carbohydrate contents were less significant. Based on these findings, the quantification of superoxide dismutase and catalase is recommended as physiological indicators of rejuvenation for *K. grandifoliola*, while superoxide dismutase is recommended for *K. senegalensis* and *K. ivorensis*. The biochemical differences observed among the materials suggest a strong relationship with the degree of physiological maturation and the expression of antioxidant mechanisms, providing important information for future research into clonal propagation and genetic improvement of *Khaya* spp. Strategies such as successive propagation cycles of rescued materials may contribute to increased adventitious rooting percentages and, consequently, to the rejuvenation/reinvigoration of tissues.

## Introduction

Species of the genus *Khaya* (Meliaceae), commonly known as African mahogany, are highly suitable for silviculture in tropical climates. They exhibit optimal growth in regions receiving more than 1000 mm of annual rainfall and with average temperatures ranging from 21 to 23 °C (Oliveira and França 2020). Over the past decade, the cultivation of *Khaya* species has attracted increasing interest from investors and rural producers due to the high commercial value of its timber, which is primarily used for furniture and interior paneling, providing greater profitability than traditional crops (Teixeira and Rodrigues 2021).

However, the main propagation method for *Khaya* species is through seeds, which has some disadvantages, including higher acquisition costs that complicate production, especially for smaller-scale growers. Additionally, seed lots may exhibit low or inconsistent germination rates, primarily due to species-specific traits, such as short storage viability and variable seed quality (Romanoski et al. 2023). Nevertheless, according to the International Tropical Timber Organization (ITTO) (ITTO 2023), the economic value of African mahogany timber has increased significantly. The price of air-dried African mahogany wood on the international market increased from €595 to €1239 m^−3^, representing a 108.24% increase, between 2009 and 2022.

Given this situation and the species’ importance for producing high-value wood, studies on alternative propagation methods are needed to enhance seedling production (Faria et al. 2024; Momolli et al. 2024). One alternative for seedling propagation, especially for multiplying selected genotypes and enabling year-round production, is vegetative propagation (Oliveira et al. 2016).

Cloning is used to increase the productivity of planted forests by homogenizing genetic material, improving wood quality, and increasing resistance to biotic stresses such as drought, water deficit, pests, and diseases (Nieri et al. 2022; Faria et al. 2024). This process also includes selecting individuals with superior provenance, characterized by better phenotypic traits, which may result from high-quality genetic material or favorable genotype-environment interaction (Costa et al. 2020).

In this manner, vegetative propagation aids in rescuing adult individuals by providing genetic material through propagules with a high degree of juvenility for seedling production, while also enabling the selection of trees with superior phenotypic traits for commercial purposes (Oliveira et al. 2016). Among vegetative propagation methods, cutting is one of the most widely used techniques, supporting intensive clonal forestry worldwide. Backed by extensive scientific research, this method represents a significant technological advancement in forestry (Xavier et al. 2021).

The rhizogenic potential or rooting ability of cuttings varies considerably among tree species and can be influenced by factors such as hormonal balance, cutting type/position, genotype, environmental conditions, cutting health, juvenility of the material, and the physiological and nutritional status of the donor plant (Hartmann et al. 2017). In other words, the success of vegetative propagation in adult tree species depends on obtaining juvenile propagules, characterized by reduced ontogenetic age (Faria et al. 2023). Ontogenetic age should not be confused with chronological age, as it is a reversible process under suitable conditions, unlike chronological age, during which cells in plant organs and tissues progressively lose their regenerative capacity as the tree matures (Xavier et al. 2021).

In woody forest species, this reversal occurs due to a juvenility gradient that intensifies toward the base of the tree (Wendling et al. 2014). The term ‘rejuvenation’ refers to this juvenility the process of reverting the plant from an adult to a juvenile stage, which directly affects the rooting potential of propagules and, consequently, seedling production (Wendling et al. 2014). Propagules can be obtained through epicormic shoots, which facilitate vegetative propagation and drive clonal forestry, with tree cutting being recommended as it stimulates the formation of new basal shoots with greater rooting capacity (Rickli et al. 2015).

Several factors can optimize the cutting process, particularly the application of auxin-based growth regulators, especially indole-3-butyric acid (IBA), which is widely recognized for its effectiveness in enhancing root formation in vegetative propagules (Sauer et al. 2013). The use of IBA can increase and accelerate the rooting rate of cuttings, as well as improve root quantity and quality, resulting in a more uniform rooting process (Oliveira et al. 2016). However, excessive auxin concentrations can inhibit root development, leading to leaf yellowing and drop, necrosis at the cutting base, and ultimately cutting mortality (Hartmann et al. 2017).

In light of this, the objective of this study was to analyze the vegetative propagation of adult individuals of *Khaya grandifoliola*, *Khaya senegalensis*, and *Khaya ivorensis* via cuttings and to investigate potential differences in tissue rejuvenation/reinvigoration through analyses of protein, carbohydrate, and antioxidant enzyme. To this end, the hypothesis was tested that seedlings of different African mahogany species can be propagated through cuttings treated with the growth regulator indole-3-butyric acid (IBA), thereby promoting a higher percentage of adventitious rooting.

## Methods

### Origin of propagules

Propagules were collected from selected trees of *Khaya grandifoliola*, *Khaya senegalensis*, and *Khaya ivorensis* species, which were felled at ten years of age in the Vale Natural Reserve, Linhares, Espírito Santo, Brazil. The propagules originated from an experimental plantation established in 2013. Seedlings of *K. ivorensis* were derived from seeds collected in plantations within the Vale Natural Reserve; seeds of *K. senegalensis* were obtained from the Selva Florestal company (Porangatu, GO), and *K. grandifoliola* seeds were acquired from Embrapa Amazônia Oriental (Belém, PA) by Atlântica Agropecuária.

The experimental plantation was established in a randomized block design, consisting of nine rectangular plots measuring 1260 m^2^ each (21 × 60 m). Treatments corresponded to three African mahogany species, planted at a 5 m × 5 m spacing, with each species represented by three replicates (totalling nine plots).

### Phenotypic data of the population

The morphological growth characteristics of the plantations were assessed for all individuals within the usable plots through a forest inventory, recording total height, commercial height, diameter at breast height (DBH), trunk form, and tree health.

Based on this assessment, the average values per species were as follows: for *Khaya grandifoliola*, the average total height was 13.3 m, commercial height was 5.8 m, and DBH was 18.7 cm. For *Khaya senegalensis*, the average total height was 10.8 m, commercial height was 4.7 m, and DBH was 20.8 cm. Finally, for *Khaya ivorensis*, the average total height was 13.7 m, commercial height was 6.0 m, and DBH was 22.8 cm. In terms of trunk form and tree health, all three species showed regular characteristics.

### Propagule collection

Based on phenotypic analyses, twelve trees from each species were selected and felled, totaling 36 individuals for the study. Prior to felling, branches with an average length of 50 cm were collected from the lower sections of each tree (approximately 5 m from the base), to minimize the influence of ontogenetic age and provide a comparative source analyses. The branches were then transferred to a climate-controlled greenhouse with regulated relative humidity (RH > 80%) and temperature (21–35 °C) equipped with an intermittent misting system using high-pressure, low-flow nozzles, which was automatically controlled by a humidistat. The branches were placed vertically in 5 L polyethylene pots filled with washed sand. After approximately 45 days, leaves from the epicormic shoots were collected and stored in an ultra-freezer at − 80 °C for subsequent biochemical and enzymatic analyses. After this stage, the same trees were felled.

The cuttings used for the adventitious rooting experiment were obtained from shoots that emerged at the base of the trunk, 10 cm above the ground level, and were collected three months after the trees had been felled. These shoots were collected and placed in containers with water at room temperature to maintain tissue turgor until they were transferred to the seedling nursery at the Federal University of Espírito Santo in Alegre—ES.

### Preparation of cuttings, experimental design and treatment application

Propagules were standardized into cuttings averaging 10 ± 2 cm in length, each with one pair of leaves reduced by half and containing two to three buds, and the basal end cut at an angle to facilitate insertion into the rooting substrate. For each species, an experiment was conducted using a completely randomized design (CRD) with three treatments and three replicates, each containing 10 cuttings, totaling 30 experimental units per species. The treatments consisted of different concentrations of indole-3-butyric acid (IBA) applied to the base of the cuttings by immersion for 15 s, as follows: T1: 0 mg L^−1^; T2: 4000 mg L^−1^; T3: 8000 mg L^−1^.

The materials were subsequently placed in 180 cm^3^ tubes, which had been disinfected by immersion in a 0.25% active chlorine sodium hypochlorite solution for 24 h, and then filled with a 1:1 (v/v) mixture of commercial substrate (Terra Nutri) and vermiculite.

The experiment was conducted in a climate-controlled greenhouse with controlled relative humidity and temperature (RH > 80%; temperature between 25 and 35 °C), equipped with an intermittent misting system using high-pressure, low-flow nozzles, irrigating seven times daily at two-hours interval.

After 60 days of staking, rooting was confirmed by the emergence of new shoots on the cuttings, which were then transferred to a shade house covered on the sides and top with 75% light-transmitting shade cloth (sombrite), featuring raised beds 80 cm above the ground and a 100-micron plastic tarp roof. After 45 days, the materials were moved to a full, where they remained for another 45 days until experimental evaluations.

**Fig. 1:**
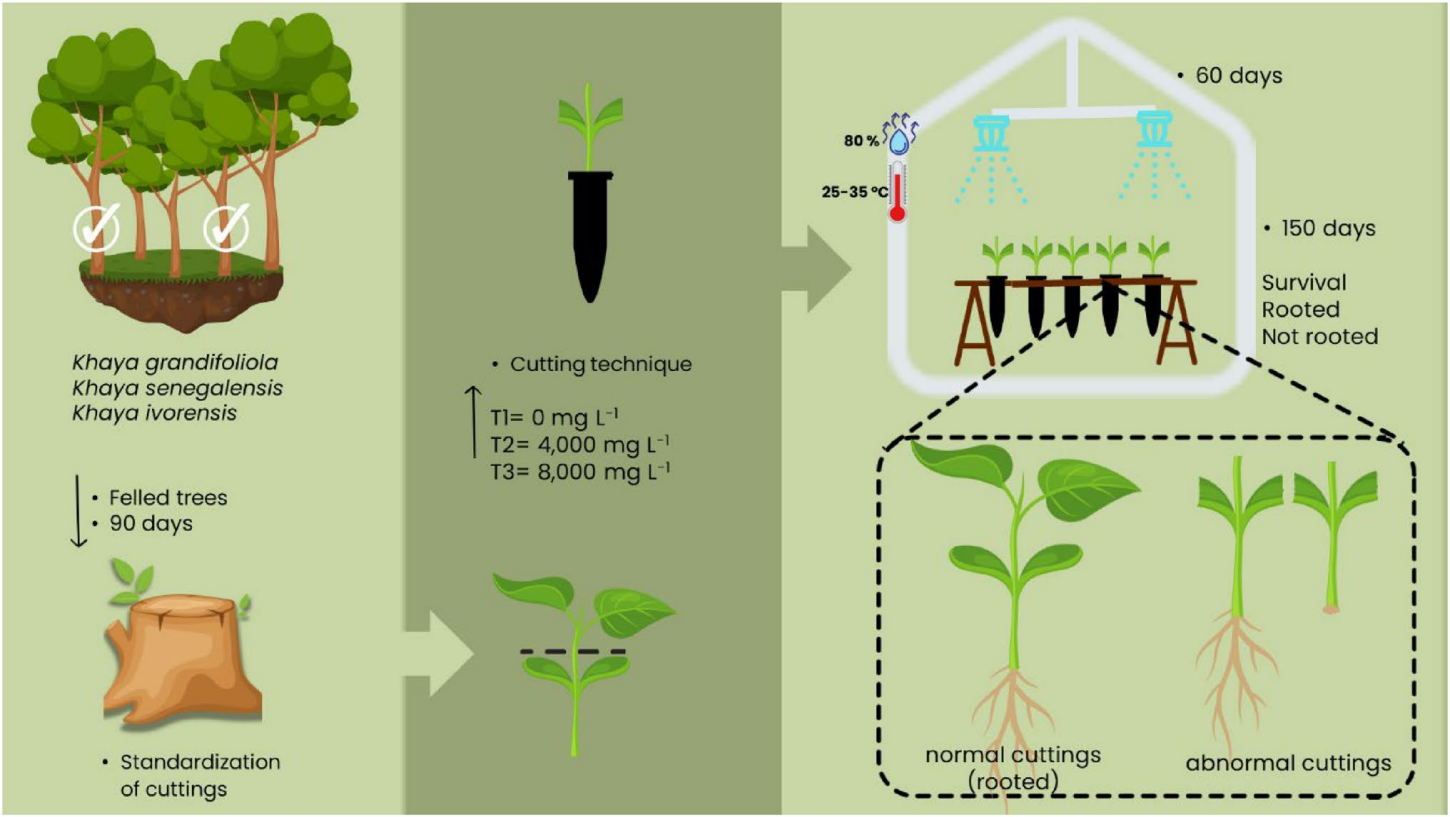
Flowchart illustrating the methodological steps of the study on adventitious rooting of cuttings derived from adult materials of *Khaya grandifoliola*, *Khaya senegalensis*, and *Khaya ivorensis*. The diagram shows the selection of parent trees, the collection of cuttings carried out 90 days after the felling of the selected trees, the establishment of the experiment in a forest nursery with the application of different concentrations of indole-3-butyric acid (IBA), and the final evaluation of survival, adventitious rooting, and the occurrence of normal and abnormal cuttings at 150 days after planting

At 150 days after staking, the survival percentage (S) (percentage of rooted cuttings plus abnormal cuttings); rooted cuttings (E) (percentage of cuttings that rooted and emitted new shoots) and abnormal cuttings (C) (percentage of living cuttings that either rooted without new shoot emission or did not root but formed callus) were determined. These steps are illustrated in the flowchart shown in Fig. 1.

### Analysis of enzymes, proteins, and carbohydrates

To assess the relationship between the adventitious rooting capacity of materials obtained through vegetative propagation by cuttings in the studied species, different plant materials were collected across the three species. For this purpose, leaf samples were collected from juvenile plants (6-month-old seedling), epicormic shoots (originating from the branches of the selected trees), adult trees(10-year-old), and recovered material (basal shoots 10 cm above the base after adventitious rooting). All leaf samples were stored in an ultra-freezer at − 80 °C until biochemical and enzymatic analyses were performed.

**Fig. 2:**
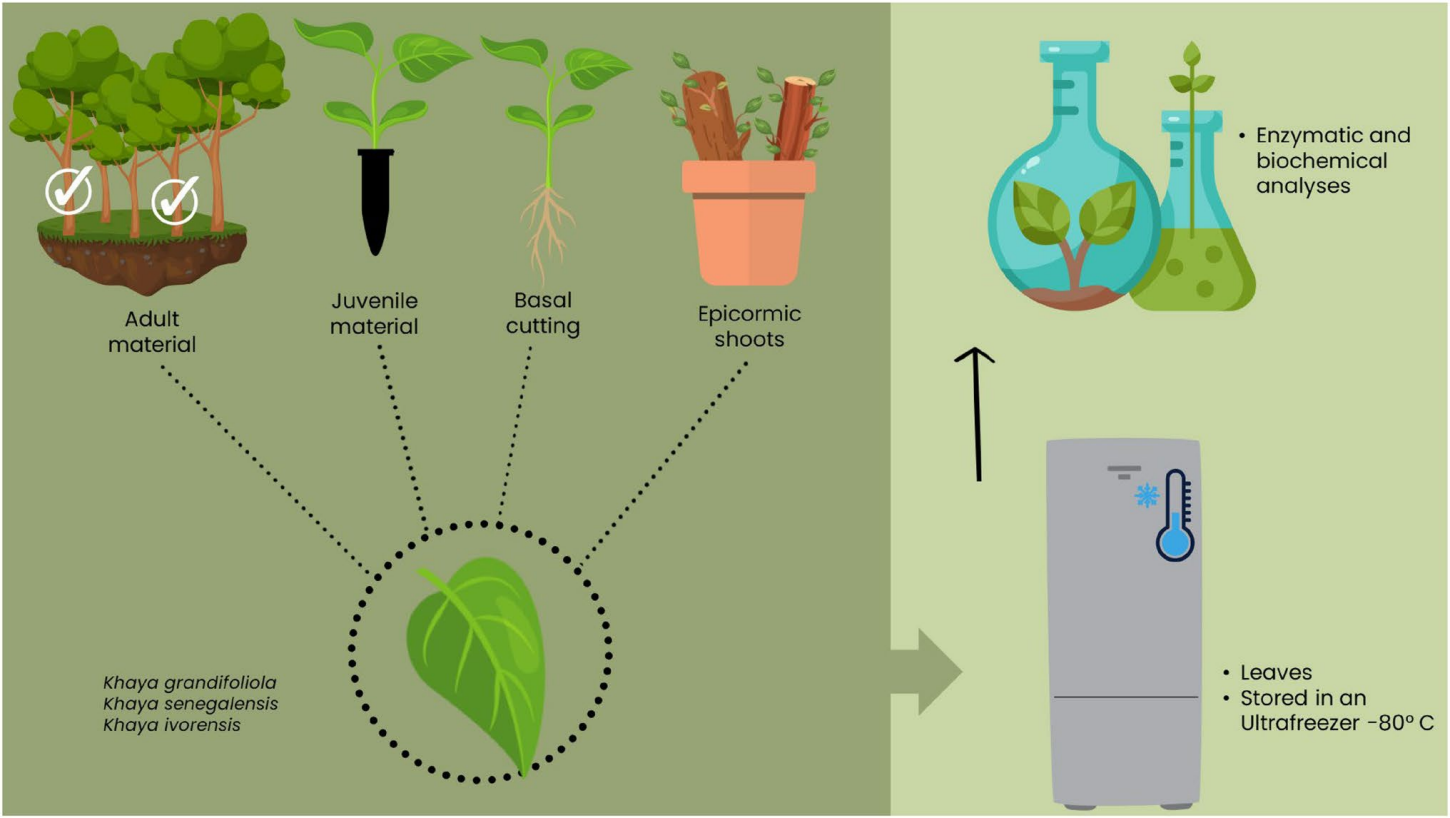
Flowchart of the methodological steps for conducting enzymatic and biochemical analyses in the investigation of tissue rejuvenation/reinvigoration in *Khaya grandifoliola*, *Khaya senegalensis*, and *Khaya ivorensis*. Four types of leaf materials were collected: juvenile (six-month-old seedlings), epicormic shoots (originating from the branches of selected trees), adult (ten-year-old trees), and rescued material (basal shoots collected 10 cm from the base after adventitious rooting). Samples were stored in an ultra-freezer at − 80 °C until analysis

From these materials, analyses of the antioxidant enzymes superoxide dismutase (SOD), catalase (CAT), and peroxidase (APX) were conducted, along with assessments of total protein and carbohydrate expression, to investigate potential indicators of rejuvenation and/or reinvigoration. The methodological steps of the enzymatic and biochemical analyses are illustrated in the flowchart in Fig. 2.

### Superoxide dismutase (SOD)

The activity of superoxide dismutase (SOD) was determined using 200 mg of leaf tissue, which was macerated in liquid nitrogen and 50% polyvinylpolypyrrolidone (PVPP). Enzymatic activity was measured by spectrophotometry according to the methods of Giannopolitis and Reis (1977) and Cembrawska-Lech et al. (2015). The reaction was conducted in chamber illuminated by a fluorescent lamp at 25 °C. For each sample, the reaction mixture consisted of 50 mM phosphate buffer (pH 7.8) + 13 mM methionine + 63 μM NBT + 0.1 mM EDTA + 1.34 μM riboflavin, totaling 1.5 ml. The measurement was performed on a spectrophotometer at 560 nm. Enzyme activity was expressed as specific activity (U SOD min^−1^ mg prot^−1^).

### Catalase (CAT)

The activity of catalase (CAT) was determined using 200 mg of leaf tissue, which was macerated in liquid nitrogen and 50% polyvinylpolypyrrolidone (PVPP). The plant samples were divided into four replicates and then homogenized in potassium phosphate buffer (100 mM, pH 7.5) containing ethylenediaminetetraacetic acid (EDTA) (1 mM), dithiothreitol (DTT) (3 mM), and 4% (w/v) PVPP. The resulting extract was centrifuged at 10,000 rpm for 30 min at 4 °C (Azevedo et al. 1998). The supernatant was collected, divided into aliquots, and frozen at − 80 °C. CAT activity was determined according to Kraus et al. (1995), with modifications by Azevedo et al. (1998), using spectrophotometry. The plant extract was added to a reaction mixture containing potassium phosphate buffer (100 mM, pH 7.5) and hydrogen peroxide (25%), prepared immediately before use. The reaction was initiated by adding 15 μl of plant extract. The activity was determined by following the decomposition of H_2_O_2_ for 1 min, by measuring changes in absorbance at 240 nm. Enzyme activity was expressed as specific activity (nmol H_2_O_2_ min^−1^ mg prot^−1^).

### Peroxidase (APX)

For peroxidase (APX) determination, 200 mg of leaf tissue was weighed and macerated in liquid nitrogen and 50% PVPP. The methodology proposed by Nakano and Asada (1981), with modifications by García-Limones et al. (2002), was adopted. Plant samples were divided into four replicates and the reaction was carried out in a medium containing phosphate buffer (100 mM, pH 7.0), EDTA (1 mM), ascorbic acid (5 mM), and hydrogen peroxide (2 mM). The reaction was initiated by adding 15 μl of plant extract to the mixture. The oxidation of ascorbic acid was measured by spectrophotometer through the decrease in absorbance at 290 nm for 1 min. Enzyme activity was expressed as specific activity (nmol ASC min^−1^ mg prot^−1^).

### Total protein extraction—TCA/acetone/phenol

For total protein analysis, leaf tissues were ground in a mortar and pestle under liquid nitrogen, with four replicates prepared for each treatment. 200 mg of macerate was used for 1.5 ml of 10% TCA in acetone. For each step, the samples were centrifuged for three minutes at 4 °C (14,000 rpm), and the supernatant was discarded. The steps consist of adding 1.5 ml of 80% methanol containing 0.1 M ammonium acetate and subsequently 1.5 ml of 80% acetone. The pellet was then dried in an oven at 50 °C for 10 min, and 0.5 ml of phenol (pH 8.0) and 0.5 ml of SDS buffer (30% sucrose, 2% SDS, 0.1 M Tris–HCl pH 8.0, 5% 2-mercaptoethanol) were added.

The samples were homogenized and incubated for five minutes, followed by centrifugation. The supernatants were transferred to new containers, 1.5 ml of methanol with 0.1 M ammonium acetate was added, and the samples were incubated at − 20 °C for 10 min. The resulting pellets were washed with 100% methanol and 80% acetone. The pellet was dissolved in urea buffer. An aliquot of each sample was used for quantification by the Bradford method (1976). Protein concentration was determined by spectrophotometry at 595 nm (Lambda 40, Perkin Elmer), using BSA ("bovine serum albumin") as a standard. Results were expressed as µg (protein) g (sample).

### Carbohydrates

To determine the content of total soluble sugars, leaf samples were dried in a forced-air oven at 60 °C until constant weight. Four replicates of 100 mg each were weighed from the dried, ground material and transferred to Erlenmeyer flasks containing 50 mL of distilled water. The flasks were placed in a water bath at 40 °C for 30 min under constant agitation. The solution was filtered through cotton, transferred to a volumetric flask, and its volume completed to 100 ml. Total soluble sugars were quantified according to Dubois et al. (1956), using 0.5 ml of the sample, 0.5 ml of phenol, and 2.5 ml of sulfuric acid. Absorbance was measured at 490 nm using a spectrophotometer. Results were expressed as g/g (sample).

### Statistical analysis

Statistical analyses were performed separately for each species. Data were tested for homogeneity of variances using Hartley’s test (p > 0.05) and for normality using the Shapiro–Wilk test (p > 0.05). When necessary, data were transformed according to the Box-Cox test. Subsequently, analysis of variance (ANOVA, p < 0.05) was performed. Based on the significance of the ANOVA, means were compared using Tukey's test (p < 0.05). All analyses were carried out using R software, version 4.3.3, with the ExpDes package (R Core Team 2025).

## Results

Statistical analyses conducted for the three species revealed similar behavior regarding survival, adventitious rooting, and occurrence of abnormalities in cuttings. Regardless of the indole-3-butyric acid (IBA) concentration used, no significant effect was observed on these variables (p > 0.05). At 150 days after cutting, the results obtained for the rescued cuttings of all three species are summarized in Table 1.

**Table 1:** Survival percentage, adventitious rooting, and abnormality in cuttings of *Khaya grandifoliola*, *Khaya senegalensis*, and *Khaya ivorensis* under different concentrations of indole-3-butyric acid (IBA) after 150 days of cutting

IBA concentration	*Khaya grandifoliola*		
	Survival	Normal cuttings (rooted)	Abnormal cuttings
	%		
0 mg L^−1^	22.1^ns^	15.5^ns^	6.6^ns^
4000 mg L^−1^	17.7	13.3	4.4
8000 mg L^−1^	13.2	6.6	6.6
Mean	17.6	11.8	5.8
CV (%)	15.8	18.5	12.9

IBA concentration	*Khaya senegalensis*		
	Survival	Normal cuttings (rooted)	Abnormal cuttings
	%		
0 mg L^−1^	27.7^ns^	12.2^ns^	15.5^ns^
4000 mg L^−1^	9.9	6.6	3.3
8000 mg L^−1^	8.8	3.3	5.5
Mean	15.4	7.3	8.1
CV (%)	21.8	25.3	13.6

IBA concentration	*Khaya ivorensis*		
	Survival	Normal cuttings (rooted)	Abnormal cuttings
	%		
0 mg L^−1^	26.6 ns	8.3^** ns**^	18.3^** ns**^
4000 mg L^−1^	11.6	5.0	6.6
8000 mg L^−1^	20.0	15.0	5.0
Mean	19.4	9.4	9.9
CV (%)	21.7	20.1	18.1

^ns^Non-significant value at 5% probability level by F-test. Data transformed by [(*n* + 0.5)/100]^0,5^, in which *n* = sampled data

*CV* coefficient of variation

**Fig. 3:**
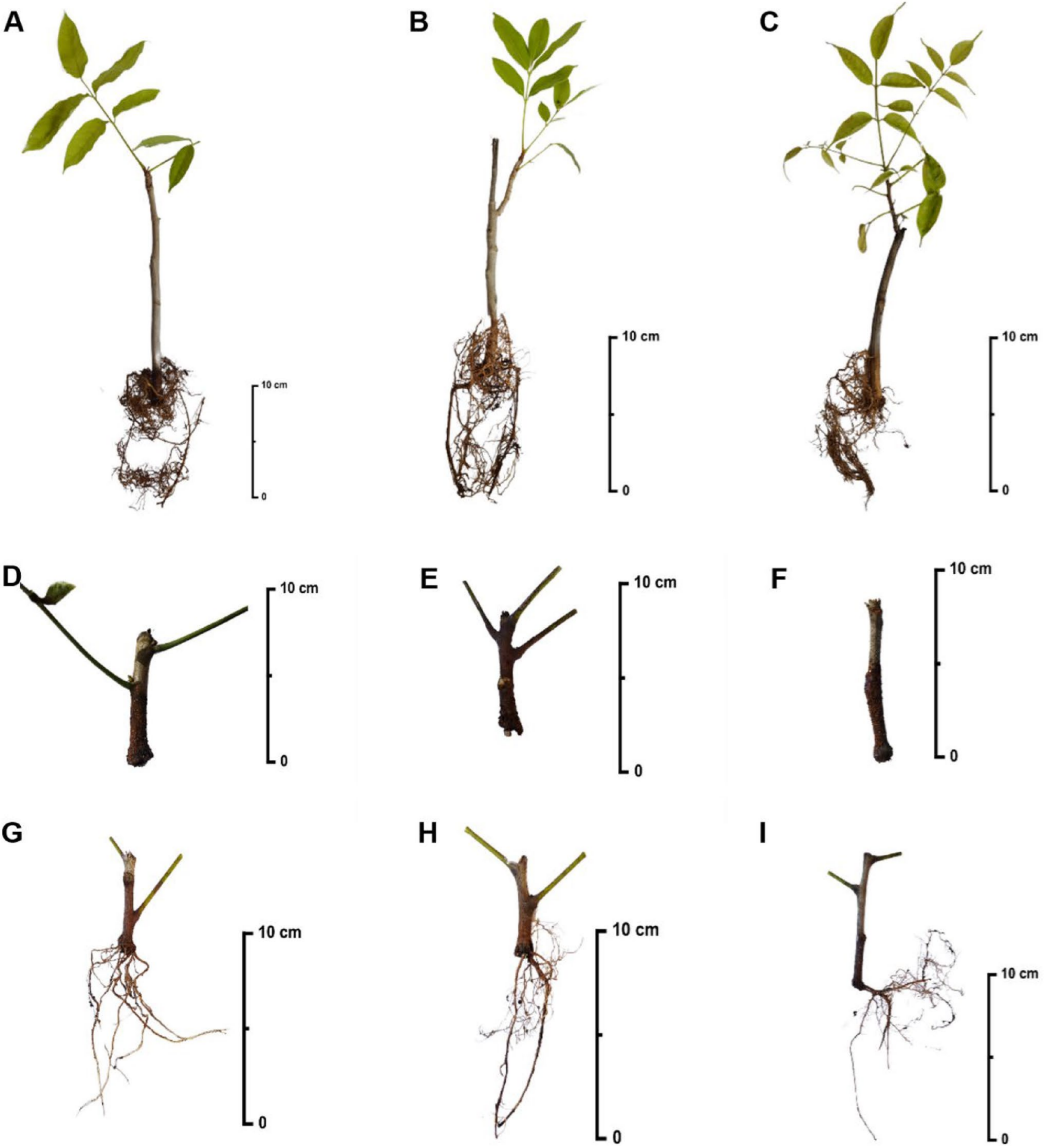
Profile of rooted cuttings of *Khaya* spp. 150 days after cutting, highlighting the presence of new shoots in **A** *Khaya grandifoliola*; **B** *Khaya senegalensis*; **C** *Khaya ivorensis*. Profile of abnormal cuttings of *Khaya* spp. 150 days after cutting showing callus formation in **D** *Khaya grandifoliola*; **E** *Khaya senegalensis*; **F** *Khaya ivorensis*; and rooted cuttings without new shoot development in **G** *Khaya grandifoliola;* **H** *Khaya senegalensis*; and **I** *Khaya ivorensis*

In *K. grandifoliola* and *K. senegalensis*, a reduction in survival and normal cutting formation was observed with increasing IBA concentrations. In *K. ivorensis*, this pattern was identified only in abnormal cutting formation. However, these variations were not pronounced, and the three tested concentrations showed no statistically significant differences among them (Table 1). Figure 3 illustrates the rooting patterns of the cuttings for each species under the different IBA concentrations, evaluated 150 days after cutting.

### Enzyme analyses, proteins, and carbohydrates

#### Khaya grandifoliola

For *Khaya grandifoliola*, all three antioxidant enzymes (SOD, CAT, and APX) exhibited similar patterns, with significantly higher activity observed in juvenile materials and cuttings rescued through propagation. SOD activity ranged from 10.20 to 11.82 min⁻^1^ mg prot⁻^1^, with no significant difference between these treatments. CAT and APX displayed the same statistical results, with the highest enzyme activity averages observed in cuttings treated with 4000 and 8000 mg L⁻^1^ of IBA. In contrast, adult materials and epicormic shoots displayed the lowest enzyme activities, with mean values around 5.66 min⁻^1^ mg prot⁻^1^ for SOD, 2.65 min⁻^1^ mg prot⁻^1^ for CAT, and 0.71 min⁻^1^ mg prot⁻^1^ for APX (Table 2).

**Table 2:** Mean activity values of superoxide dismutase (SOD), catalase (CAT), peroxidase (APX), proteins (PROT), and total carbohydrates (CARB) in leaf samples obtained from different propagation materials of *Khaya grandifoliola*

Trataments	SOD	CAT	APX	PROT	CARB
	U SOD min^−1^ mg prot^−1^	nmol H_2_O_2_ min^−1^ mg prot^−1^	nmol ASC min^−1^ mg prot^−1^	µg (prot) g (sample)	g/g (sample)
T1 (adult material)	2.5365 c	2.3624 c	0.6606 c	3.3287 b	0.0018 d
T2 (juvenile material)	11.7018 a	7.6159 b	6.2401 b	14.0299 a	0.0035 c
T3 (epicormic shoots)	8.7933 b	2.9558 c	0.7500 c	2.6087 b	0.0018 d
T4 (basal shoots 0 mg L^−1^ AIB)	11.8202 a	7.9160 b	7.2616 a	1.0498 c	0.0072 a
T5 (basal shoots 4000 mg L^−1^ AIB)	10.2100 ab	9.4150 ab	7.2914 a	0.8795 c	0.0055 b
T6 (basal shoots 8000 mg L^−1^ AIB)	11.2621 a	10.4552 a	7.3212 a	1.2201 c	0.0049 b

T1. Adult material: selected trees at 10 years old; T2. Juvenile material: 6-month-old seedlings; T3. Epicormic shoots: branches from 10-year-old adult trees; T4. Rooted cuttings from basal shoots (0 mg L⁻^1^ of IBA); T5. Rooted cuttings from basal shoots (4000 mg L⁻^1^ of IBA); T6. Rooted cuttings from basal shoots (8000 mg L⁻^1^ of IBA).

The quantification of total proteins (PROT) and soluble sugars (CARB) revealed significant differences among the different types of material. For total proteins, significantly higher levels were observed in juvenile material, followed by adult material and epicormic shoots, while the lowest means were recorded in all cuttings treated with different IBA concentrations. In contrast, soluble sugars showed an opposite pattern, with significantly higher levels in material propagated through cuttings and the lowest means in adult, juvenile, and epicormic shoot material (Table 2).

### Khaya senegalensis

For *K. senegalensis*, enzymatic activities showed distinct results among the materials. CAT was the enzyme that most clearly distinguished the rejuvenation/reinvigoration of the materials, with its highest expression in juvenile material (3.53 min⁻^1^ mg prot⁻^1^) and the lowest in adult material (0.5426 min⁻^1^ mg prot⁻^1^). In contrast, SOD and APX did not exhibit the same pattern as CAT, making it more difficult to differentiate adult from juvenile materials based on their enzymatic expression, as both showed the highest statistical averages across treatments (Table 3).

**Table 3:** Mean activity values of Superoxide dismutase (SOD), Catalase (CAT), Peroxidase (APX), proteins (PROT), and carbohydrates (CARB) in leaf samples obtained from different materials of *Khaya senegalensis*

Trataments	SOD	CAT	APX	PROT	CARB
	U SOD min^−1^ mg prot^−1^	nmol H_2_O_2_ min^−1^ mg prot^−1^	nmol ASC min^−1^ mg prot^−1^	µg (prot) g (sample)	g/g (sample)
T1 (adult material)	3.1800 d	0.5426 e	0.6344 c	5.3801 c	0.0035 d
T2 (juvenile material)	3.5800 d	3.5316 a	0.3074 c	26.7928 a	0.0048 c
T3 (epicormic shoots)	10.9803 b	2.8623 b	1.8426 b	7.5829 c	0.0036 d
T4 (basal shoots 0 mg L^−1^ AIB)	16.2788 a	1.2614 d	2.4318 ab	19.3182 b	0.0079 b
T5 (basal shoots 4000 mg L^−1^ AIB)	15.3882 a	1.4325 cd	3.2133 a	14.8788 b	0.0083 ab
T6 (basal shoots 8000 mg L^−1^ AIB)	5.2421 c	1.8548 c	2.8933 ab	17.0985 b	0.0087 a

T1. Adult material: selected trees at 10 years old; T2. Juvenile material: 6-month-old seedlings; T3. Epicormic shoots: branches from 10-year-old adult trees; T4. Rooted cuttings from basal shoots (0 mg L⁻^1^ of IBA); T5. Rooted cuttings from basal shoots (4000 mg L⁻^1^ of IBA); T6. Rooted cuttings from basal shoots (8000 mg L⁻^1^ of IBA)

For SOD, cuttings without IBA application and those treated with 4000 mg L⁻^1^ of IBA showed the highest averages levels (16.27 and 15.38 min⁻^1^ mg prot⁻^1^, respectively), both statistically superior to the other treatments. For APX, all materials propagated via cuttings, regardless of IBA application, showed the highest statistical averages, with values around 2.84 min⁻^1^ mg prot⁻^1^ (Table 3).

The analysis of total proteins (PROT) presented the highest statistical average in the juvenile material (26.79 µg prot g), with the cuttings showing values close to this, but statistically lower. For total carbohydrates (CARB), the highest averages were expressed cuttings treated with 4000 and 8000 mg L⁻^1^ of IBA, with the highest averages being 0.0083 and 0.0087 g/g, respectively. In contrast, the adult material and the epicormic shoots presented the lowest values for both proteins and total carbohydrates, being statistically inferior to the juvenile material and cuttings (Table 3).

#### Khaya ivorensis

Considering the expressions of SOD and CAT, the juvenile material obtained the highest average results, being statistically superior to the others. On the other hand, APX showed the opposite trend, with the juvenile material presenting the lowest average value (0.9524 nmol ASC min⁻^1^ mg prot⁻^1^). The epicormic shoots showed some of the lowest SOD and CAT activities, with CAT values being statistically similar to those of the adult material (Table 4).

**Table 4:** Mean activity values of Superoxide dismutase (SOD), Catalase (CAT), Peroxidase (APX), proteins (PROT), and carbohydrates (CARB) in leaf samples obtained from different materials studied of *Khaya ivorensis*

Trataments	SOD	CAT	APX	PROT	CARB
	U SOD min^−1^ mg prot^−1^	nmol H_2_O_2_ min^−1^ mg prot^−1^	nmol ASC min^−1^ mg prot^−1^	µg (prot) g (sample)	g/g (sample)
T1 (adult material)	8.0800 b	1.9983 d	1.8204 b	18.8699 a	0.0016 c
T2 (juvenile material)	16.8763 a	7.3735 a	0.9524 c	19.3613 a	0.0023 c
T3 (epicormic shoots)	6.0877 c	2.7688 cd	1.4649 b	3.1444 d	0.0038 b
T4 (basal shoots 0 mg L^−1^ AIB)	7.4067 b	5.9418 b	1.0922 c	10.679 c	0.0052 a
T5 (basal shoots 4000 mg L^−1^ AIB)	8.7256 b	3.7123 c	2.2944 a	15.8185 b	0.0052 a
T6 (basal shoots 8000 mg L^−1^ AIB)	7.7336 b	3.4036 c	1.6933 b	8.0818 c	0.0053 a

T1. Adult material: selected trees at 10 years old; T2. Juvenile material: 6-month-old seedlings; T3. Epicormic shoots: branches from 10-year-old adult trees; T4. Rooted cuttings from basal shoots (0 mg L⁻^1^ of IBA); T5. Rooted cuttings from basal shoots (4000 mg L⁻^1^ of IBA); T6. Rooted cuttings from basal shoots (8000 mg L⁻^1^ of IBA)

In terms of total proteins, *K. ivorensis* displayed a different pattern compared to the other species, with statistically higher results in the adult and juvenile materials, while the cuttings and epicormic shoots showed significantly lower levels. Total carbohydrates levels were statistically higher in the cuttings, regardless of IBA application, with an average value of 0.0048 g/g, while the adult and juvenile materials were statistically similar and inferior to the others, with an average result of 0.0019 g/g of the sample (Table 4).

**Fig. 4:**
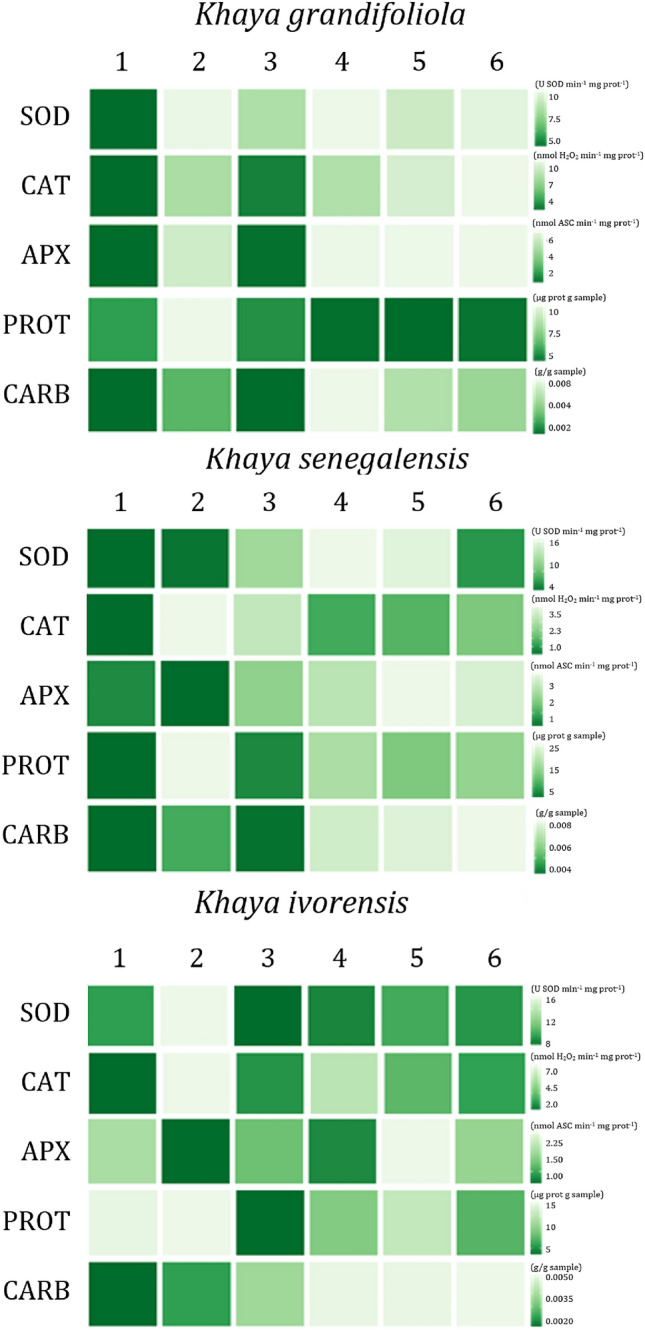
Heatmap showing the correlations between biochemical and enzymatic variables based on Pearson’s correlation coefficient, with significance at 5%. The rows of the dendrogram represent the variables analysed, while the columns correspond to the treatments applied. Color variations in green indicate expression levels, with more intense shades associated with lower enzymatic activities and lighter shades indicating higher expression levels

In summary, the graphical representation of the enzymatic analyses, proteins, and total carbohydrates is shown in Fig. 4. In this dendrogram, the rows represent the different analyses performed, while the columns correspond to the treatments applied. The graphical representation of the heatmap displays the expression values through colors that vary according to their intensity. To visualize the different levels of expression, the color green was assigned, with the more intense shade representing lower enzymatic activity, proteins, and total carbohydrates, and the less intense shade representing higher levels of expression. This representation emphasizes that treatments involving adult-origin materials exhibit lower activity, whereas juvenile materials demonstrate higher activity (Fig. 4).

## Discussion

Considering that the propagation of forest species is commonly done through the adventitious rooting of cuttings, the effects of material maturation become a major challenge for the success of the propagation of superior genotypes. This is mainly due to the decline in rooting competence as the ontogenetic age or maturation of the source material increases (Varas et al. 2023). Therefore, higher rooting percentages are often associated with the juvenility of the material and the age of the parent plant (Chang et al. 2024), as well as factors such as the position of shoot collection, physiological vigor of the propagule, and genetic material differences (Xavier et al. 2021). In this study, basal cutting propagation from felled trees achieved adventitious rooting across all three species. However, the rooting percentages were relatively low, not exceeding 30%.

Nevertheless, *Khaya* species are recognized as difficult to propagate through adventitious rooting, as no commercial clones of selected individuals have been developed to date. Given that African mahogany has a medium-term cultivation cycle, of approximately twenty years, investigating clonal propagation parameters, which remain scarce in the literature, is essential to achieving productivity gains in the forestry of these species.

The few studies available in the literature on vegetative propagation of *Khaya* species have predominantly used juvenile material for adventitious rooting (Barbosa Filho et al. 2016; Barroso et al. 2018; Silva et al. 2020), which may not be the best strategy when aiming to select individuals for future clonal tests with superior genotypes of interest. The most widely adopted technique for clonal propagation of African mahogany is grafting, which allows the union of tissues both within and between species of the genus. However, grafting requires trained professionals to perform it properly and presents low success rate depending on the age of the target individuals (Barbosa Filho et al. 2016; Opoku et al. 2019).

Among vegetative propagation techniques in forest species, cutting is the most widely used. In this study, we evaluated whether IBA application could influence the adventitious rooting of basal cuttings. After 150 days of cultivation, however, no significant differences were observed between the tested concentrations for any of the three species. In percentage terms, *K. grandifoliola* and *K. senegalensis* exhibited the highest absolute values of adventitious rooting and new shoot emergence without IBA application, whereas K*. ivorensis* showed the opposite trend. Some authors have suggested that IBA application at the base of cuttings does not influence adventitious rooting of the materials, as this mechanism is due to the endogenous production of auxin in each plant (e.g., endogenous auxin synthesis) (Dias et al. 2015; Hartmann et al. 2017). These variations can be explained by the genetic differences among species, as well as differences in endogenous auxin levels and other rooting cofactors in the plant (Ross et al. 2024), even within the same genus.

Thus, to investigate potential indicators of rejuvenation/reinvigoration in rescued adult materials, this study examined three antioxidant enzymes associated with adventitious rooting in the cuttings of three *Khaya*species, as well as total protein and carbohydrate contents. Enhancing the antioxidant defense system is a key adaptive response in plants under oxidative stress (Fatma et al. 2021; Tavu et al. 2025). SOD and CAT act together to convert superoxide into oxygen and water. SOD serves as the first line of defense by converting superoxide into hydrogen peroxide, while CAT then converts hydrogen peroxide into water and oxygen. APX complements CAT's action, especially in organelles like chloroplasts, where H_2_O_2_ is generated during the photosynthesis process (Fatma et al. 2021).

Once the shoots are removed from the mother plant, the supply of nutrients and water is interrupted. Improving their stress resistance and reducing the time required for root formation are crucial for the survival of the cuttings. Antioxidant enzymes play not only an important role in a plant's antioxidant defense but also affect the formation and development of adventitious roots (Sun et al. 2023a, b). In this study, the synthesis of antioxidant enzymes SOD, CAT, and APX in the three species (*K. grandifoliola*, *K. senegalensis*, and *K. ivorensis*) varied among treatments, forming two groups: the first group comprised juvenile materials and cuttings from the base with 0, 4000, and 8000 mg L⁻^1^ IBA, and the second group comprised adult materials and epicormic shoots. This behavior can be explained by the active growth of juvenile tissues, requiring higher protein synthesis, cell division, and other metabolic processes that may be associated with specific enzymatic activities (Khan et al. 2022).

Among the enzymes analyzed, CAT showed similar activity in all materials rescued via cuttings with base shoots to juvenile materials, regardless of IBA application and species. On the other hand, adult tissues and epicormic shoots showed equivalent enzymatic activity in general terms, likely due to the fact that epicormic shoots were obtained from branches of adult plants. This difference may be attributed to the juvenility gradient of the materials, which is greater towards the base of the tree, suggesting a reversal process from the adult to the juvenile stage in materials via cuttings, with a direct impact on the rooting potential of the propagules (Faria et al. 2023).

Regarding total protein concentrations, significant variation was observed between the adult and juvenile materials of *K. grandifoliola* and *K. senegalensis* after 150 days of cutting. This difference can be explained by the stress adaptation response, in which there is an increase in the activity of proteolytic enzymes responsible for breaking down reserve proteins (Fan and Jespersen 2025). Considering the use of materials from different sources in this study, and the clustering of treatments into two groups as previously discussed, it can be inferred that the materials represent "adult" and "juvenile" categories. In this context, the literature suggests that protein activity tends to be lower in adult materials and higher in juvenile materials, as the latter require intense cell division for their development and growth (Taiz et al. 2017).

In relation to carbohydrates, an inversely proportional relationship with total proteins was observed in the three species for juvenile materials. One possible explanation is related to germination, a process in which starch is broken down into soluble sugars through the enzymes alpha-amylase and maltase. These soluble sugars are consumed during seed respiration, resulting in reduced total carbohydrate content (Taiz et al. 2017; Zhang et al. 2025). In this study, an inversely proportional relationship between proteins and carbohydrates was observed in the materials of *K. grandifoliola* and *K. ivorensis*, indicating that when protein activity was at its lowest level, carbohydrates showed their highest potential in each treatment. This dynamic may be attributed to the greater amount of energy reserves present in adult materials. On the other hand, in basal cuttings, where adventitious rooting occurs, carbohydrate content plays a crucial role, as rhizogenesis requires a significant amount of energy. Under high-temperature conditions, the synthesis of sucrose and starch is increased to support the energy demands of the rooting process (Taiz et al. 2017).

However, variations in protein concentrations, sugars, and particularly in the activity of the enzymes SOD, CAT, and APX, support the hypothesis that cuttings undergo an initial period of intense stress due to the removal of the parent plants, the cutting preparation process, the cutting environment, and the IBA treatment as performed in this research. Thus, this study demonstrates the viability of genetic rescue through the cutting technique, suggesting the need for further research. Most studies on vegetative propagation of species in the genus *Khaya* are limited to the use of juvenile material (Barbosa Filho et al. 2018; Barroso et al. 2018; Azevedo et al. 2021). In contrast, the present study is innovative in exploring cloning from rescued adult genotypes, enabling the multiplication of individuals that express superior phenotypic traits under field conditions. Although the adventitious rooting percentages obtained are still considered moderate for all three species, it is expected that successive rooting cycles will promote a progressive rejuvenation/reinvigoration of tissues, gradually increasing these percentages.

Moreover, there remains a lack of in-depth studies investigating the physiological mechanisms and factors responsible for variations in adventitious root formation (Wang et al. 2025). The biochemical analyses conducted in this study revealed distinct enzymatic responses among the evaluated species, indicating the presence of species-specific physiological strategies with potential to improve the efficiency of vegetative propagation.

A relevant approach regarding the limitations of rejuvenation/reinvigoration in plant tissues may be associated with restrictions imposed by epigenetic mechanisms, which are still not fully understood (Wang et al. 2023). Among these mechanisms, DNA methylation, chromatin remodeling, histone modifications, and small RNA-regulated changes stand out, all of which play roles in controlling the cellular developmental state in plants (Liu et al. 2024). However, despite advances in understanding these processes, the present study focused on generating technical information aimed at vegetative propagation, with the objective of optimizing the mechanisms involved in inducing adventitious rooting in adult *Khaya* spp. individuals. This is the first applied study in this context, stablishing a basis for future research in clonal propagation and genetic improvement of these forest species of great importance for the high-quality timber sector. This study represents a first step towards discoveries that could enhance the selection of genotypes of interest in timber production and enable the production of clonal seedlings from selected trees.

## Conclusion

Basal cuttings obtained from felled trees induced adventitious rooting in all studied species, although rooting percentages remained relatively low, not exceeding 30%.

In general, basal cuttings treated with 0, 4000 and 8000 mg L⁻^1^ of IBA exhibited enzymatic activity levels of superoxide dismutase, catalase, and peroxidase comparable to those observed in juvenile seed-derived material.

For studies focused on tissue rejuvenation/reinvigoration and related metabolic activities, it is recommended to quantify superoxide dismutase and catalase enzymes in *K. grandifoliola*, while for *K. senegalensis* and *K. ivorensis*, only superoxide dismutase is advised.

Heatmap analysis indicated that protein and total carbohydrates levels were the least responsive indicators for distinguishing tissue differences among African mahogany species. These findings highlight the need for further research to elucidate specific biochemical pathways and better understand adventitious rooting in adult plant materials of these species.

## Data Availability

The datasets generated during and/or analyzed during the current study are available from the corresponding author on reasonable request.
